# Supplementary material for: Distribution and associations for antimicrobial resistance and antibiotic resistance genes of Escherichia coli from musk deer (Moschus berezovskii) in Sichuan, China
Source: PLoS One. 2023 Nov 27;18(11):e0289028. doi: 10.1371/journal.pone.0289028 (PMC10681177; doi:10.1371/journal.pone.0289028)
Supplement: Attachment — Submitted filename: Response to Reviewers.docx [file pone.0289028.s003.docx]

Response to Reviewer 1 Comments

**Response to Reviewer:**

Thank you very much for your time and thoughtful comments, many of which have been incorporated into the revised manuscript (Tracked Version). Detailed responses are as follows.

Question 1: The authors mention that antibiotic-resistant *E. coli* carrying ARGs in musk deer’s feces that are discharged into the environment may become pollutants for nature. How about some specific suggestions on the implementation of solve this problem?

**Response 1:** Thanks for your comments. We add some suggestions in our revised manuscript (Lines 249-251): We suggest that the feces forest musk deer should be disinfected to reduce the environmental pollution caused by antibiotic-resistant *E. coli*.

Question 2: Were the isolates also tested for colistin/polymyxin? In the current scenario, it would be interesting to search for the mcr genes in this study.

**Response 2:** Thanks for your comments. AMC, CN, CRO, AK, ENR, FFC and FOX have been used in Sichuan Institute of Musk Deer Breeding for disease treatment and control. Colistin/polymyxin was not used in the two farms. As the reviewer suggestion, colistin/polymyxin resistance is a topic for scientists in recent years. In our ongoing research, we conducted a preliminary experiment to detect *mcr* gene. However, colistin resistance gene *mcr* was not detected in all *E. coli* isolated from musk deer (data not showed in our current manuscript). Moreover, *mcr* gene was also not detected in *E. coli* isolated from other captive wild animals (monkey, black bear, golden pheasant, and silver pheasant) in our laboratory (data not published).

Question 3: Were PCR positive and negative controls performed in their study?

**Response 3**: Thanks for your comments. We have set up a negative control, but antibiotic resistance genes (ARGs) amplify do not need positive control in general. There have many researches amplify ARGs without positive control (1, 2). In our study, we used primers derived from references and the length of the PCR products was determined and compared to the 2000 DNA Marker (Sangon Biotech, Shanghai, China) during gel electrophoresis. Notably, for the confirmation of each ARG, the PCR products were purified and sequenced by Sangon Biotech (Shanghai, China). Then, the DNA sequence data were confirmed via the GenBank online BLAST software. All the above analysis was to ensure the accuracy of PCR products.

Question 4: In Table 1 and Table 4, unit % is given at the top, so there is no need to duplicate it in the content.

**Response 4**: Thanks for your comments. We delete % at the top of Table 1 and Table 4 in our revised manuscript.

Question 5: Line 68-69, Please list detailed species that have detected AMR associated with ARGs.

**Response 5**: Thanks for your comments. We add detailed species (honeybees, poultry and pig) that have detected AMR associated with ARGs in our revised manuscript (Lines 69-70).

Question 6: Please analyze the difference of drug resistance between Dujiang Yan and Barkam in the discussion part.

**Response 6**: Thanks for your comments. The difference rate of antibiotic resistant between Dujiang Yan and Barkam was added in discussion parts (Lines 230-236): The antibiotic resistance rates of Dujiang yan and Barkam are similar, but the antibiotic resistance rates of Barkam to TE, KZ, CXM, SAM and CIP are significantly higher than Dujiang yan.

Response to Reviewer 2 Comments

**Response to Reviewer:**

Thank you very much for your time and thoughtful comments, many of which have been incorporated into the revised manuscript (Tracked Version). Detailed responses are as follows.

Question 1: In Sample collection, how to know the feces samples from each animal accurately? Please explain methods of collection samples carefully, and to ensure each samples correspondence to each animal.

**Response 1:** Thanks for your comments. The samples were collected by professional musk deer’s breeder before feeding periods. Breeders can identify musk deer individually by morphological characteristics to ensure that each fecal sample comes from an individual musk deer. As soon as the musk deer defecated, the feces were collected immediately. The process was added in our revised musk deer (line 79-81): Fresh fecal specimens (approximately 10 g) from each musk deer were collected immediately by feeders after defecation on the ground and then quickly transferred into individual 50-mL plastic containers.

Question 2: The results found that there has some difference in AMR/MDR between Dujiang Yan and Barkam, and why this phenomenon was not mentioned in the discussion?

**Response 2:** Thanks for your comments. Some discussions were added in our revised musk deer (line 232-236): The rate of MDR from Barkam is higher than that from Dujiang Yan, indicateds antibiotic resistant in Bakarm is more severe and more caution should be exercised when choosing antibiotics. The antibiotic resistance rates of Bakarm to TE, KZ, CXM, SAM and CIP are significantly higher than that from Dujiang Yan, which may relate to different living environments

Question 3: In this study, *Escherichia coli* strains from clinically healthy captive musk deer carrying a large number of ARGs, which make a little difficult to understanding this phenomenon. I suggest in the discussion the author should consider add some information to explain this phenomenon.

**Response 3:** Thanks for your comments. We have added some explanations and related references to the phenomenon of *E. coli* carrying ARGs in healthy animals for better understanding by readers in the discussion (lines 248-249): ARGs carried in clinically healthy animal (non-human primates, giant pandas and waterfowl birds) pose a potential threat to the surrounding environment.

Question 4: Table 8 may not be well expressed, which need revised concise to better understanding. Meanwhile, only one cassette array (*dfrA17*-*aadA5*) was identified. Have the results been repeated three times to make sure the accuracy of their results?

**Response 4:** Thanks for your comments. Table 8 was reorganized in our revised manuscript. As the reviewer mentioned, gene cassette was detected three times in our experiment to make sure the accuracy of our results, and only one cassette array (*dfrA17*-*aadA5*) was identified.

Reference:

1.	Gorecki A, Musialowski M, Wolacewicz M, Decewicz P, Ferreira C, Vejmelkova D, et al. Development and validation of novel PCR primers for identification of plasmid-mediated colistin resistance (mcr) genes in various environmental settings. Journal of hazardous materials. 2022;425:127936.

2.	Rumky J, Kruglova A, Repo E. Fate of antibiotic resistance genes (ARGs) in wastewater treatment plant: Preliminary study on identification before and after ultrasonication. Environmental research. 2022;215(Pt 1):114281.
